# Comparison of cytokine expression and disease severity between plasma cell-dominant and eosinophil-dominant patients in chronic rhinosinusitis with nasal polyps

**DOI:** 10.1186/s13223-024-00896-6

**Published:** 2024-05-21

**Authors:** Yu-Tsai Lin, Ming-Hsien Tsai, Yan-Ye Su, Shun-Chen Huang

**Affiliations:** 1 Department of Otolaryngology, Kaohsiung Chang Gung Memorial Hospital, Chang Gung University College of Medicine, Kaohsiung, Taiwan; 2 Kaohsiung Chang Gung Head and Neck Oncology Group, Cancer Center, Kaohsiung Chang Gung Memorial Hospital, Kaohsiung, Taiwan; 3 Department of Otolaryngology, Kaohsiung Chang Gung Memorial Hospital, Kaohsiung, School of Medicine, College of Medicine, National Sun Yat-sen University, Kaohsiung, Taiwan; 4 Department of Anatomic Pathology, Kaohsiung Chang Gung Memorial Hospital and Chang Gung University, Kaohsiung, Taiwan; 5 Department of Otolaryngology, Kaohsiung Chang Gung Head and Neck Oncology Group, Cancer Center, Kaohsiung Chang Gung Memorial Hospital, 123 Ta-Pei Road, Niao-Song District, Kaohsiung, 833 Taiwan; 6 Department of Anatomic Pathology, Kaohsiung Chang Gung Memorial Hospital, Chang Gung University, 123 Ta-Pei Road, Niao-Song District, Kaohsiung, 833 Taiwan

**Keywords:** Chronic rhinosinusitis, Nasal polyps, Eosinophil, Plasma cell, Interleukin-6

## Abstract

**Purpose:**

Chronic rhinosinusitis with nasal polyps (CRSwNP) is a heterogeneous disease characterized by inflammation of the nasal and sinus mucosa. The inflammatory patterns may differ among patients, leading to different subtypes based on the dominant inflammatory cell type. This study aimed to compare the differences in cytokine expression and disease severity between plasma cell-dominant and eosinophil-dominant subtypes in patients with CRSwNP.

**Methods:**

This study included 53 CRSwNP patients and 19 control subjects who did not have asthma or a history of cigarette smoking. The expression of cytokines and inflammatory cells was assessed via enzyme-linked immunosorbent assay (ELISA) and immunohistochemistry, respectively.

**Results:**

Among the cytokines analyzed, only IL-6 was significantly different between the two subtypes. A greater proportion of mast cells and IgE cells was present in plasma cell-dominant CRSwNP patients than in eosinophil-dominant group. For the three disease severity scores (LMK-CT, TPS and SNOT-22), objective scores (LMK-CT and TPS) were greater in the eosinophil-dominant CRSwNP group, while the opposite result was shown for the subjective score (SNOT-22). Additionally, the percentage of plasma cell-dominant cells was significantly positively correlated with disease severity according to the TPS and SNOT-22 scores.

**Conclusions:**

Our data revealed that plasma cell-dominant inflammation, a subtype of type 2 CRS, was significantly correlated with subjective disease severity. The study also highlights the role of IL-6, IgE and mast cells as distinguishing factors between eosinophil-dominant and plasma cell-dominant CRSwNP. This information could be useful for clinical diagnosis and personalized treatment.

**Supplementary Information:**

The online version contains supplementary material available at 10.1186/s13223-024-00896-6.

## Introduction

Chronic rhinosinusitis (CRS) is a prevalent medical condition that involves chronic inflammation in the paranasal sinus mucosa. It has a substantial impact on various aspects, including socioeconomic burden, quality of life and healthcare costs globally [1, 2]. When nasal polyps (NPs) are present and inflammation in the nasal airway and sinuses persists for more than 12 weeks, the condition is referred to as chronic rhinosinusitis with nasal polyps (CRSwNP) [3]. CRS is a complex condition characterized by clinical, pathological and immunological diversity in which inflammation plays a key role and is characterized by chronic inflammation of the sinonasal mucosa with inflammatory infiltration of lymphocytes, monocytes, plasma cells and eosinophils. Additionally, pathological changes occur in the mucoepithelial structures of the nasal epithelium [4, 5].

The presence of eosinophilic inflammation is indeed a hallmark of type 2 inflammation; nevertheless, the prevalence of this type 2 inflammatory endotype can vary among different populations. In Western countries, a significant majority of CRSwNP patients, 73–87%, are classified as having a type 2 inflammatory endotype characterized by eosinophilic infiltration. This type of inflammation is especially common among white populations, with eosinophilic inflammation present in 65–90% of nasal polyp cases [6–8]. In Asian populations, the prevalence of the type 2 inflammatory endotype has historically been lower, ranging from 17 to 61%, although recent research has indicated that the proportion of patients with eosinophilic chronic rhinosinusitis (eCRS) is increasing in Asian regions [9–11]. Studies have shown changing inflammatory patterns in nasal polyps from patients with CRSwNP in northern China over the past 4–5 decades, with an increased proportion of eosinophil-dominant CRSwNP cases [10]. In northern Taiwan, approximately 64% of CRSwNP cases have been identified as the eosinophilic endotype [11].

eCRS and CRSwNP are believed to develop predominantly through type 2 immune responses [12]. Allergens, which include pollens and mites, enter the nasal mucosa and induce a subsequent immune response in which dendritic cells capture allergens and transmit information to naive CD_4_-positive T cells, which differentiate into Th2 cells under stimulation by IL-4 to produce IL-4, IL-5, and IL-13. It has been reported that IL-4 and IL-6 are factors that stimulate the differentiation of B cells into allergen-specific IgE-producing plasma cells, which subsequently produce IgE [13, 14]. Recent studies have reported that the immune system, particularly B cells, plasma cells and antibodies, is highly active within the nasal mucosa of CRSwNP patients [15]. Baird et al. indicated that in CRSwNP, 63% of patients exhibited an inflammatory predominance characterized by lymphoplasmacytic infiltration [16]. The 2020 European Positioning Paper on Rhinosinusitis (EPOS2020) [3] indicated the complexity and heterogeneity of type 2 chronic rhinosinusitis (type 2 CRS) among patients. This finding suggests that not only does the intensity of inflammation vary among individuals but also that there may be distinct subtypes characterized by the enhancement of specific aspects of the immune pathway. The abovementioned subtypes involve the activation of different immune cells, such as mast cells, eosinophils, and plasma cells. These cell types play a role in the immune response, and their enhanced activity in specific subtypes may contribute to the diverse clinical manifestations observed in type 2 CRS. In our clinic, we recently observed increased plasma cell infiltration in the stroma of nasal polyps in patients with CRS by hematoxylin and eosin (H&E staining), although the distribution of plasma cell dominance remains unclear. Therefore, this study analyzed nasal polyp tissue from CRSwNP patients in the Taiwanese population to identify and quantify type 2 inflammatory cells, such as eosinophils, plasma cells and mast cells, and their associated cytokine expression patterns as well as to assess the relationship of these cells with disease severity.

## Materials and methods

### Subjects

**Fig. 1:**
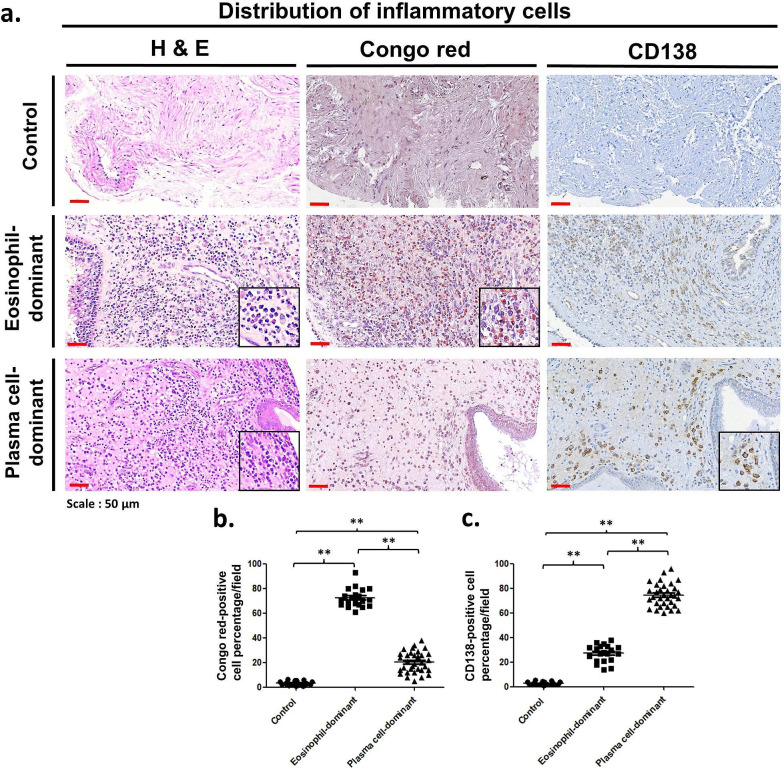
(**a**) Identification of eosinophil and plasma cell expression in the nasal tissues of study subjects. H&E staining (left panel) revealed florid inflammatory cells in the stroma of the eosinophil-dominant CRSwNP and plasma cell-dominant CRSwNP groups. Congo red staining (middle panel) highlighted many eosinophils in the eosinophil-dominant CRSwNP group. CD138 IHC (right panel) revealed that plasma cells were especially abundant in the plasma cell-dominant CRSwNP group. Scale bar = 50 μm. The numbers of (**b**) Congo red-positive cells and (**c**) CD138-positive cells were quantified via Image-Pro Plus 6.0. **p < 0.01. H&E: hematoxylin and eosin

The study included a total of 72 subjects from the Department of Otolaryngology of Kaohsiung Chang Gung Memorial Hospital in Taiwan enrolled between September 2017 and August 2023. Nasal polyps from 20 eosinophil-dominant CRSwNP patients and 33 plasma cell-dominant CRSwNP patients who received endoscopic sinus surgery were assessed. A control group of biopsy tissues from the middle nasal mucosae of 19 subjects who underwent septomeatoplasty for relief of nasal obstruction due to nonallergic chronic rhinitis was obtained. Individuals with previous sinonasal surgery, nasal tumors, or other sinonasal diseases were excluded. CRS was diagnosed on the basis of the criteria outlined by the EPOS2020 [3]. Several criteria were applied to exclude certain individuals from the study. The exclusion criteria included patients with allergic fungal rhinosinusitis (AFRS) and aspirin-exacerbated respiratory disease (AERD); patients who had taken systemic corticosteroids or immunomodulating drug therapy within 12 weeks before surgery; patients who had underlying immunologic gastrointestinal, renal, endocrine or skeletal disorders that might affect immune responses; patients with recurrent CRS or asthma; and patients who were cigarette smokers. For the classification of the eosinophil or plasma cell-dominant CRSwNP, the eosinophil was determined by Congo Red staining. The plasma cell was analyzed by the immunohistochemistry of CD138. The study used Image-Pro Plus 6.0 (Media Cybernetics, LP, USA) to quantify the staining of certain cells by counting the number of positive cells per area (20,000 μm²/field) in stained sections, while immunohistochemistry intensity was determined based on specific staining percentages as follows: staining percentage ≥ 60% Congo Red-positive cells as the eosinophil-dominant group and staining percentage ≥ 60% CD138-positive cells as the plasma cell-dominant group. Six random fields were counted per section (Fig. 1). This study was approved by the Institutional Review Board of Chang Gung Medical Foundation (approval numbers 201701016B0, 201900340B0 and 202202108B0).

### Measurement of cytokines

The levels of the cytokines IL-4, IL-5, IL-6, IL-13, IL-17 and tumor necrosis factor alpha (TNF-α) were determined using the Bio-Plex Human Cytokine Group Assay Kit (Bio-Rad Laboratories, Hercules, CA, USA). Antibodies specific for human IL-4, IL-5, IL-6, IL-13, IL-17 and TNF-α were immobilized or coated on 96-well plates, ensuring that the cytokines in the samples and standards were bound to these immobilized antibodies. After the cells were washed, they were flushed in triplicate for unbound biotinylated antibody, and horseradish peroxidase (HRP)-conjugated streptavidin was pipetted into the wells. 3,3’,5,5’-Tetramethylbenzidine (TMB) was added to the wells, and the color developed in proportion to the amount of cytokines. A stop solution was added to halt color development, and the intensity of the developed color (yellow) was measured at 450 nm using a Bio-Plex suspension array system. The data were analyzed using Bio-Plex Manager software version 6.0 (Bio-Rad Laboratories).

### Tissue microarray

Tissue microarrays were developed with multiple tissue samples on a single microscope slide using the SIDSCO-TMA70 system (Scientific Integration Design Service Corp., Kaohsiung, Taiwan). The tissue sections on the microarrays were immunostained with the following primary antibodies: anti-CD138 (1:100, #36-2900; Thermo Fisher Scientific, MA, USA) and anti-IgE (1:100, ab75673; Abcam, Cambridge, UK), followed by incubation with a rabbit polyclonal antibody. All slides were mounted with xylene-based mounting medium and scanned at 400× using Olympus OlyVIA software (Olympus, Tokyo, Japan). The staining intensity was determined using Image-Pro Plus software (version 6.0; Media Cybernetics, LP, USA). The immunohistochemistry intensity was analyzed as follows: staining percentage ≤ 25% of cells positive for weak staining, 26–50% of cells positive for mild to moderate staining, and > 51% of cells positive for strong staining.

### Measurement of eosinophils and mast cells

From the paraffin-embedded blocks of the selected subjects, 4 μm thick sections were cut and stained with conventional H&E stain. Effective eosinophil and mast cell expression was confirmed by histological analysis and Congo Red- and toluidine blue-stained paraffin sections, respectively. The resulting stain showed that the eosinophil granules were bright red and the nuclei were dark blue, although toluidine blue should stain the mast cells dark violet and the background light blue.

### Measurement of nasal polyp symptom severity scores

For assessment of nasal polyp symptom severity, (1) sinus computed tomography (CT) scores were calculated based on the Lund-Mackay scale (LMK-CT) [17], which includes grading each paranasal sinus from 0 to 2 based on the level of opacification, with total scores ranging from 0 to 24 points, where a higher score indicates greater severity of the disease. (2) The total nasal endoscopic polyp score (TPS) was used to evaluate the extent and severity of nasal polyps through nasal endoscopy, with each nostril scored on a scale of 0 to 4 and the total score being the sum of the left and right nostril scores, with a range of 0–8; a higher TPS indicated more severe nasal polyps [18]. (3) The 22-item Sinonasal Outcome Test (SNOT-22) was translated into Chinese for native Chinese speakers, and this patient-reported outcome questionnaire was used to estimate the impact of nasal polyps on a patient’s quality of life while patients recalled their experience over a period of two weeks. This questionnaire allows patients to provide self-reported data, with a maximum possible score of 110 points; higher scores indicate greater impact of nasal polyps on a patient’s quality of life [19].

### Statistical analysis

Statistical analysis was performed using GraphPad Prism 5.0 software (GraphPad Software, Inc., La Jolla, CA, USA). Differences among groups were tested by one-way analysis of variance with Bartlett’s test for post-hoc testing and a chi-square test. Unpaired Student’s *t* tests were used to compare two groups. The correlation of disease severity scores was determined by Pearson’s correlation test. The results are presented as the means and standard errors as the means ± SEs. A *p* value less than 0.05 (*p* < 0.05) was considered to indicate a significant difference.

## Results

### Patient demographics

Twenty eosinophil-dominant CRSwNP patients, 33 plasma cell-dominant CRSwNP patients, and 19 control subjects were included in this study. The demographic information for each group, including the presence of atopy and peripheral eosinophils (%), is described in Table 1. There was no difference in sex or age between the groups. The serum IgE concentration was greater in the eosinophil-dominant CRSwNP and plasma cell-dominant CRSwNP patients than in the control group (*p* < 0.0001); moreover, there was a significantly greater serum IgE concentration in the plasma cell-dominant CRSwNP group than in the eosinophil-dominant CRSwNP group (*p* < 0.0019). For staining, hematoxylin and eosin (H&E), Congo red, and immunohistochemical (IHC) staining were performed with an anti-CD138 antibody, and the distributions of the eosinophils and plasma cells are presented in Fig. 1. In the left panel, H&E staining revealed that inflammatory cells infiltrated the flora in the stroma of the eosinophil-dominant CRSwNP and plasma cell-dominant CRSwNP groups but were almost nonexistent in the control group. The middle panel with Congo red staining shows that eosinophils were abundant in the eosinophil-dominant CRSwNP patients; in the right panel, CD138-positive cells were more abundant in the plasma cell-dominant CRSwNP patients than in the eosinophil-dominant CRSwNP patients (Fig. 1a). After quantification, Fig. 1b shows that Congo red-positive cells were significantly more common in the eosinophil-dominant CRSwNP patients (*p* < 0.01), while CD138-positive cells were more highly expressed in the plasma cell-dominant CRSwNP patients than in the eosinophil-dominant CRSwNP patients (Fig. 1c, *p* < 0.01).

**Table 1:** Clinical demographics of the subjects in this study

		**Subjects**		
Variables	**Control** *(n = 19)*	Eosinophil-dominated **CRSwNP** *(n = 20)*	Plasma cell-dominated **CRSwNP** *(n = 33)*	*p* value
Gender (male), n (%)	11 (57.9)	14 (77.8)	25 (75.0)	0.3067
Age (years), Mean ± SE	37.9 ± 2.9	50.3 ± 2.8	45.9 ± 2.7	0.3482
Peripheral eosinophil (%), Mean ± SE	3.2 ± 0.7	4.6 ± 0.7	3.3 ± 0.4	0.3260
Serum IgE level (IU/L), Mean ± SE	51.3 ± 7.7	89.4 ± 22.9	197.4 ± 40.0	< 0.0001*
Methodologies used				
Tissue ELISA (n)	19	20	33	-
Tissue IHC (n)	19	20	33	-

∗Significance was considered at *p* < *0.05*; CRSwNP: chronic rhinosinusitis with nasal polyps; IgE: immunoglobulin E; ELISA: enzyme-linked immunosorbent assay; IHC: immunohistochemistry

### IgE and mast cells in eosinophil- and plasma cell-dominant CRSwNP

**Fig. 2:**
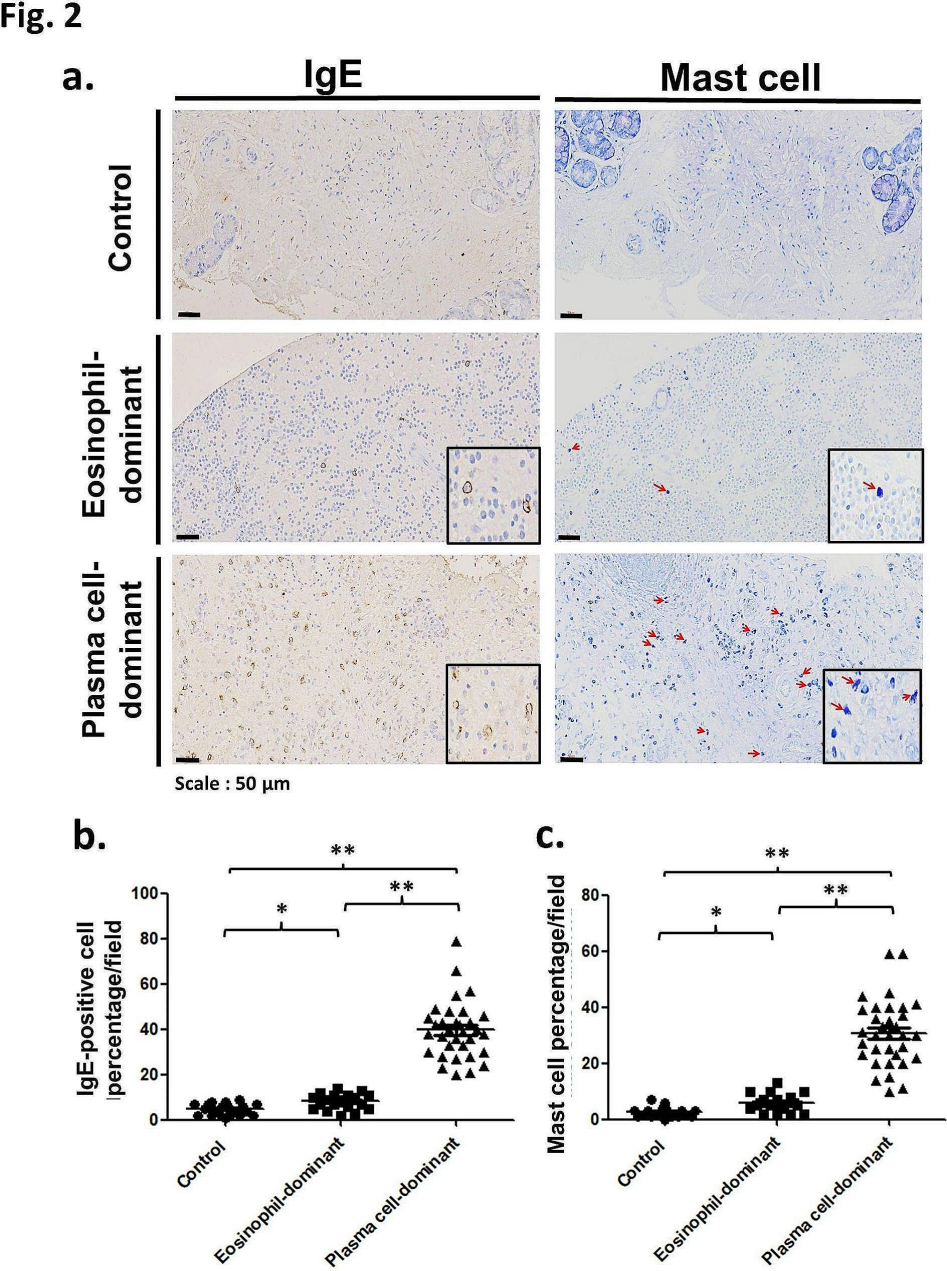
(**a**) IgE (IHC, left panel) and mast cell (Toluidine blue stain, right panel) expression in nasal tissues of control and study subjects. Scale bar = 50 μm. The quantification of (**b**) IgE-positive cells and (**c**) mast cells was performed with Image-Pro Plus 6.0. Red arrow: toluidine blue staining. *p < 0.05; **p < 0.01. IgE: immunoglobulin E

The expression of IgE in all nasal mucosa tissues was determined by IHC. There were few IgE-positive cells in the control group; however, slightly increased in the eosinophil-dominant CRSwNP, but were significantly augmented in the plasma cell-dominant CRSwNP group (Fig. 2a, left panel). However, the number of IgE-positive cells was still significantly greater in the eosinophil-dominant CRSwNP group than in the control group according to statistical analysis (*p* < 0.05; Fig. 2b), while the number of IgE-positive cells was significantly greater in the plasma cell-dominant CRSwNP group; thus, the *p* value between the control and plasma cell-dominant CRSwNP groups and between the eosinophil-dominant CRSwNP and plasma cell-dominant CRSwNP groups was less than 0.01 (Fig. 2b).

In the right panel of Fig. 2a, we used toluidine blue staining to identify mast cells; similarly, the number of mast cells was low in the control group, slightly increased in the eosinophil-dominant CRSwNP group, and markedly increased in the plasma-dominant CRSwNP group. After quantification, the number of mast cells was significantly different between the control and eosinophil-dominant CRSwNP groups (*p* < 0.05), between the control and plasma-dominant CRSwNP groups (*p* < 0.01), and between the eosinophil-dominant CRSwNP and plasma-dominant CRSwNP groups (*p* < 0.01) (Fig. 2c).

### Cytokines and clinical assessments of disease severity scores in eosinophil- and plasma cell-dominant CRSwNP patients

The levels of cytokines, such as IL-5, -6, -13, -17 and TNF-α in whole nasal polyp tissues were measured via ELISA, as shown in Table 2. IL-4 (short half-life) concentrations were nondetectable in all groups (data not shown). The levels of IL-5, -6, -13, -17 and TNF-α in the control group were 13.02 ± 1.29, 102.41 ± 25.94, 10.03 ± 1.44, 6.07 ± 2.00 and 35.03 ± 5.84,  respectively. The amount of IL-6 in the plasma cell-dominant CRSwNP group was significantly greater than that in the eosinophil-dominant CRSwNP and control groups. While the expression levels of IL-5, -13, -17 and TNF-α in the eosinophil-dominant CRSwNP group and in the plasma cell-dominant CRSwNP group were significantly greater than those in the control group (both *p* < 0.001; data not shown), there were no significant differences between these expression levels in the eosinophil-dominant CRSwNP and plasma cell-dominant CRSwNP groups (Table 2). A comparison of clinical disease severity scores between the eosinophil-dominant group and plasma cell-dominant group in CRSwNP patients is presented in Table 3, with the results showing that the measurable scores for LMK-CT and TPS in the eosinophil-dominant CRSwNP group were significantly greater than those in the plasma cell-dominant CRSwNP group (*p* = 0.0258 and 0.0233, respectively). Conversely, there were significantly greater measurable scores for SNOT-22 in the plasma cell-dominant CRSwNP group than in the eosinophil-dominant CRSwNP group (Table 3).

**Table 2:** Comparison of cytokines between eosinophil-dominant and plasma cell-dominant patients with CRSwNP.

	CRSwNP groups			
**Cytokines (**Mean ± SE)	Eosinophil-dominant (*n* = 20)	Plasma cell-dominant (*n* = 33)		*p* value
**IL-5**	60.77 ± 5.40	50.09 ± 3.77		0.2217
**IL-6**	205.78 ± 26.48	293.62 ± 27.56		0.0434*
**IL-13**	38.04 ± 5.10	38.30 ± 3.04		0.9293
**IL-17**	25.35 ± 5.52	30.64 ± 4.37		0.2709
**TNF-α**	71.29 ± 15.63	57.42 ± 8.84		0.5509

CRSwNP: chronic rhinosinusitis with nasal polyps; IL: interleukin; TNF-α: tumor necrosis factor alpha. *p < 0.05 was considered to indicate a statistically significant difference

**Table 3:** Comparison of clinical assessments of disease severity scores between eosinophil-dominant and plasma cell-dominant CRSwNP patients

	CRSwNP groups			
**Scores** **(**Mean ± SE)	Eosinophil-dominant (*n* = 20)	Plasma cell-dominant (*n* = 33)	*p* value	
**LMK-CT**	17.50 ± 1.12	14.33 ± 0.89	0.0445*	
**TPS**	6.11 ± 0.32	4.94 ± 0.34	0.0388*	
**SNOT-22**	65.72 ± 3.72	77.18 ± 1.40	0.0233*	

CRSwNP: chronic rhinosinusitis with nasal polyps; LMK-CT: Lund-Mackay computed tomography; TPS: total nasal endoscopic polyp score; SNOT-22: 22-item sinonasal outcome test; *p < 0.05 was considered to indicate statistical significance

**Fig. 3:**
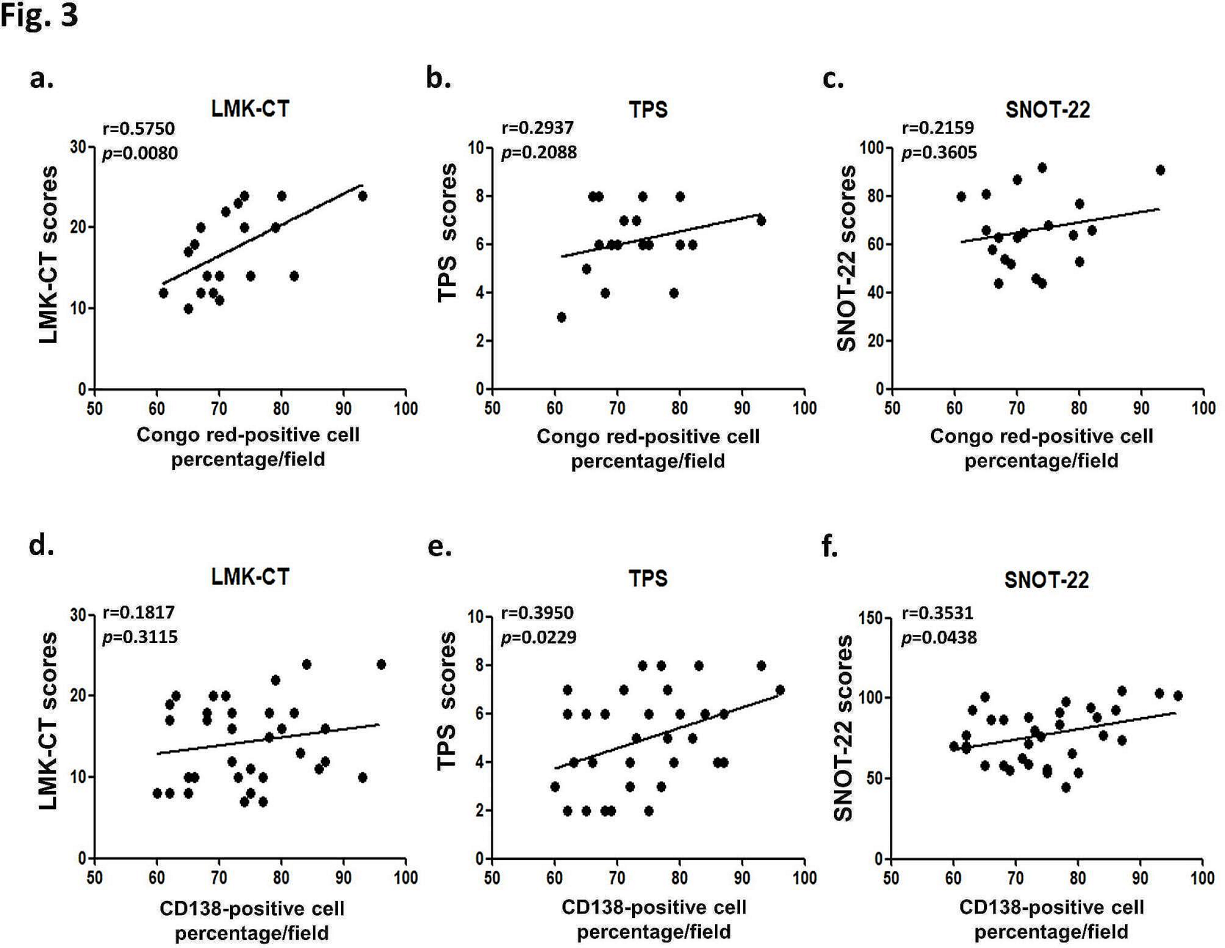
The correlation between the eosinophil-dominant (the percentage of Congo red-positive cells) or plasma cell-dominant (the percentage of CD138-positive cells) phenotype and the three clinically measurable disease severity scores (LMK-CT, TPS and SNOT-22) was analyzed by the Pearson R test. (**a~c**) Correlations between the percentages of Congo Red-positive cells and (**a**) LMK-CT, (**b**) TPS, and (**c**) SNOT-22 expressions. (**d~f**) Correlations between the percentages of CD138-positive cells and (**d**) LMK-CT, (**e**) TPS, and (**f**) SNOT-22 expressions. LMK-CT: Lund-Mackay computed tomography; TPS: total nasal endoscopic polyp score; SNOT-22: 22-item sinonasal outcome test; *p < 0.05 was considered to indicate a statistically significant difference

The correlation between the eosinophil-dominant or plasma cell-dominant CRSwNP group and disease severity is presented in Fig. 3. Pearson correlation analysis revealed a significant positive correlation between the percentage of Congo red-positive cells in the eosinophil-dominant group and the measurable LMK-CT score (*r* = 0.5750; 95% CI = 0.1133–0.6541) (Fig.3 a∼c). Moreover, there was a significant correlation between the percentage of CD138-positive cells in the plasma cell-dominant group and the measurable scores for the TPS and SNOT-22 (*r* = 0.3950; 95% CI = 0.01245- 0.1560; and *r* = 0.3531; 95% CI = 0.01881-1.266, respectively) (Fig. 3d∼f).

## Discussion

Many studies have indicated that eosinophils play an important role in CRS clinical characteristics. Eosinophil-dominant inflammatory cell infiltration is reportedly found in 60 to 90% of CRSwNP patients in Europe and the United States, where the definition of eosinophil-dominant infiltration requires a threshold of 5 to 10 or more eosinophils per field (total magnification of 400×) to classify tissue samples as eosinophil dominant [20]. In Japan, the number of eosinophils per field (total magnification of 400×) requires a greater number of eosinophils per field, either 70 or more or 200 or more [13]. In our cases, the threshold for considering tissue samples as eosinophil dominant or plasma cell dominant was when the number of eosinophils or plasma cells per field (total magnification of 400×) reached 60% or more.

In the past, Asian CRSwNP patients had different profiles of inflammatory immune cells, often exhibiting less eosinophilic and more neutrophilic inflammation than their Caucasian counterparts [21]; however, more recently, there has been a shift in the inflammatory patterns of CRSwNP patients in Asia. The incidence of eosinophil-dominant inflammation has significantly increased in CRSwNP patients in this region [8, 9, 13, 21]. In Japan, there is evidence that the incidence of noneosinophilic chronic rhinosinusitis (noneCRS) is decreasing, while that of eCRS is increasing [13]. Similarly, in South Korea, the incidence of eosinophilic chronic rhinosinusitis with nasal polyps (eCRSwNP) was significantly greater in 2011 (62.6%) than in earlier years (47.7%) [9], while in China, the proportion of eCRSwNP also significantly increased from 59.1 to 73.7% over an 11-year period [22]. These findings are consistent with our findings that approximately 75% of Taiwanese CRSwNP patients exhibit type 2 inflammation.

Early studies used immunohistochemical staining to identify CD20 cells (a marker for B cells) in nasal polyp tissue Sect. [23]. Recently, flow cytometry staining for CD19 and CD138 (a plasma cell marker) revealed significantly elevated levels of both B cells and plasma cells in nasal polyps [24]. Our study used Congo Red staining to identify eosinophils and CD138 staining to identify plasma cells in nasal polyps. This analysis revealed that 29% of the nasal polyps were eosinophil dominant and 47% were plasma cell dominant, indicating a greater prevalence of plasma cells in those polyps. We excluded seven CRSwNP patients (10%) with neutrophil-dominant inflammation (Supplementary Fig. S1) and ten patients with mixed eosinophil-dominant and plasma cell-dominant patterns (14%), allowing us to focus on the specific type 2 inflammatory profiles; moreover, patients with a history of cigarette smoking were also excluded. Cigarette smoking was associated with increased levels of IL-17 and type 3 inflammation [25, 26]. Although the reason for this difference has yet to be confirmed, the noticeable increase in the proportion of plasma cells in southern Taiwan CRSwNP patients could be due to specific local conditions, including air pollution, a humid environment, or a particular ethnic genotype. These factors could contribute to the difference in the inflammatory signatures of CRSwNP patients in this region compared to those in other regions.

A wealth of evidence indicates that Th2 cytokines control the major components of the inflammatory response, including IgE isotype switching, mucus production, and the recruitment and activation of eosinophils [27, 28]. The cytokines were detected, and although IL-4 (short half-life) concentrations were undetectable in all groups, IL-5, IL-6, IL-13, IL-17 levels were markedly greater in the eosinophil- and plasma cell-dominant CRSwNP groups than in the control group; in particular, the IL-6 level was significantly greater in the plasma cell-dominant CRSwNP group. A previous report indicated that IL-6 could be a major cytokine in nasal polyp physiopathology [29], possibly playing an important role in promoting significant increases in plasma cells and antibodies. IL-6 was originally named B-cell stimulation factor 2 (BSF-2) due to its ability to induce class switch recombination in B cells, as well as to differentiate B cells into plasma cells [30, 31]; consequently, our data indicated that the numbers of IgE cells and mast cells in the plasma cell-dominant CRSwNP group were greater than those in the eosinophil-dominant CRSwNP group, where objective clinical assessments of disease severity, such as TPS and LMK-CT, were significantly greater in the eosinophil-dominant CRSwNP group than in the plasma cell-dominant CRSwNP group, while the results were reversed in terms of subjective disease severity measurable scores for SNOT-22. A positive relationship was found by Pearson correlation between the percentage of plasma cell-dominant cells and disease severity measurable scores for the TPS and SNOT-22. Wu et al. [32] showed that tissue eosinophil infiltration in the sinus mucosa and Lund-Mackay scores were greater in recurrent nasal polyp patients who required revision surgery than in those who did not. Taken together, these findings suggest that IL-6 might stimulate B-cell differentiation, which then transforms into allergen-specific IgE-producing plasma cells with high IgE production. Binding of IgE to mast cells triggers the release of chemical mediators, particularly eosinophil chemotactic factors, and this release in response to various antigens might result in eosinophilic inflammation [13, 14].

CRS is considered a complex disease, and both genetic and environmental factors contribute to its pathogenesis [33]. Several studies have examined cytokine profiles in CRS patients in Taiwanese populations [11, 34, 35], but fewer reports have investigated the predominance of inflammatory cells in CRSwNP patients in such populations and correlated this difference with disease severity. In conclusion, our study’s results suggest that, in more than 75% of CRSwNP patients in Taiwan, there is a type 2 inflammation-dominant profile, including approximately 29% of eosinophil- and 47% of plasma cell-dominant patterns that are correlated with IL-6 and subjective disease severity according to our questionnaire. We believe that the observation of objective severity scores was certainly greater in the eosinophil-dominant CRSwNP is important as well as. Nevertheless, it is suggested that the findings of this study be considered to contribute to the diagnostic criteria for CRSwNP in clinical settings, as should the findings of plasma cell-dominant CRSwNP be considered accordingly, although further validation studies are needed. Additionally, these findings have implications for personalized treatment approaches based on the immune profile of CRSwNP patients.

### Electronic supplementary material

Below is the link to the electronic supplementary material.

Supplementary Material 1
