# Supplementary figures and images for: Comparison of cytokine expression and disease severity between plasma cell-dominant and eosinophil-dominant patients in chronic rhinosinusitis with nasal polyps
Source: Allergy Asthma Clin Immunol. 2024 May 21;20:34. doi: 10.1186/s13223-024-00896-6 (PMC11110371; doi:10.1186/s13223-024-00896-6)

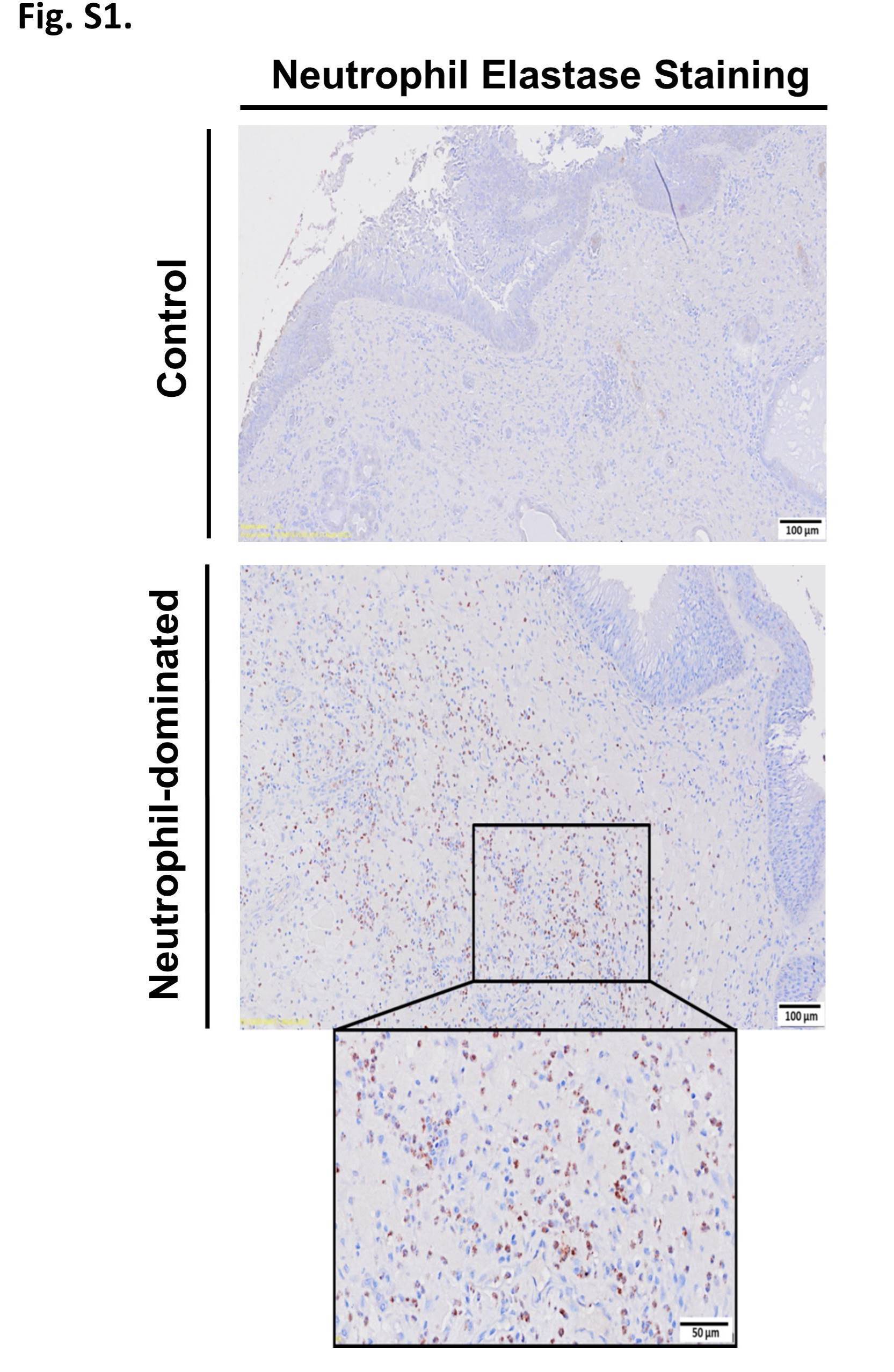

Supplement: Supplementary file 1 — Supplementary Material 1 [file 13223_2024_896_MOESM1_ESM.jpg]
